# Involvement in daily life activities from the perspectives of children and young people with childhood‐onset disabilities: A scoping review

**DOI:** 10.1111/dmcn.70228

**Published:** 2026-03-12

**Authors:** Vera C Kaelin, Kinga Quaaden, Helena Lindgren, Mats Granlund, Christine Imms

**Affiliations:** ^1^ Department of Computing Science Umeå University Umeå Sweden; ^2^ Institute of Health Sciences, School of Health Sciences OST ‐ Eastern Switzerland University of Applied Sciences St. Gallen Switzerland; ^3^ Insitute of Occupational Therapy, School of Health Sciences Zurich University of Applied Sciences Winterthur Switzerland; ^4^ Institute for Applied Mathematics and Physics, School of Engineering Zurich University of Applied Sciences Winterthur Switzerland; ^5^ CHILD, Department of Social Work Jönköping University Jönköping Sweden; ^6^ Healthy Trajectories Child and Youth Disability Research Hub, Department of Paediatrics The University of Melbourne Parkville VIC Australia

## Abstract

**Aim:**

To gain a comprehensive understanding of participation, particularly the construct ‘involvement’, in daily life activities from the perspective of children and young people with childhood‐onset disabilities.

**Method:**

We conducted a scoping review of literature from PubMed, CINAHL, and PsycINFO. We included articles if they examined the experiences of involvement in activities from the perspective of children and young people with childhood‐onset disabilities, used qualitative methods, and were published in peer‐reviewed journals after 2001. Data (e.g. age, narratives on involvement) from included studies were extracted, mapped, and thematically synthesized.

**Results:**

Of 5241 articles, 30 met the inclusion criteria. We identified six concepts representing the experience of involvement in activities from the perspective of children and young people with childhood‐onset disabilities. One concept reflected a continuum of inner dedication or investment in activities, ranging from non‐involvement to involvement overload, which can be understood as the in‐the‐moment experience of involvement, representing the core of the involvement experience. Five additional ideas captured how children and young people process, interpret, and reflect on their experiences of involvement.

**Interpretation:**

Results can inform paediatric rehabilitation measures and interventions focused on involvement. Future research should explore children and young people's involvement in undesirable activities and whether the concepts of involvement vary by context (e.g. country, activity setting) and personal factors (e.g. age, type of disability).

## Graphical abstract

This scoping review aimed to understand the construct ‘involvement’ in daily life activities from the perspective of children and young people with childhood‐onset disabilities. We identified six conceptual ideas, including a continuum of inner dedication or investment in‐the‐moment, and five others reflecting how children and young people process, interpret, and reflect on their involvement experiences.

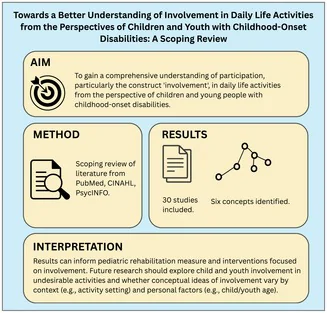

**Abbreviation**
fPRC: family of participation‐related constructs

**What this paper adds**

- This study enhances the understanding of participation by defining ‘involvement’ as a multidimensional construct comprising six concepts.
- One concept reflects a continuum of inner dedication or investment in an activity.
- Five concepts highlight how children and young people process, interpret, and reflect on their involvement experiences.
- Most research in this area focuses on involvement in pleasant or preferred activities.

Participation is a key (re)habilitation and early childhood intervention outcome, which has been defined in the International Classification of Functioning, Disability and Health,^1^ as ‘involvement in a life situation’. This definition has been further conceptualized by Imms et al.^2^, ^3^ in the family of participation‐related constructs (fPRC) framework, defining participation as both ‘attendance’ and ‘involvement’. ‘Attendance’ is described as ‘being there’^2^ and can be objectively measured in terms of attendance frequency or the diversity of activities.^2^, ^3^ ‘Involvement’ is characterized as ‘the experience of participation while attending’^2^ and, as such, cannot be fully observed from an external perspective.^2^, ^3^, ^4^ Participation‐focused paediatric rehabilitation services can, therefore, focus on children and young people's attendance in activities, their involvement in activities, or on both. In the fPRC, the transactions among participation and related constructs (i.e. the child's environment/context, activity competencies, sense of self, and preferences) are considered key influences on participation experiences. The fPRC was developed on the basis of paediatric rehabilitation research; however, the participation concept is universal^5^ and the framework has been applied across diverse settings and populations, including adults.^6^, ^7^

While the dimension ‘attendance’ is well understood and commonly measured in participation‐focused research and paediatric rehabilitation practice,^8^ currently there is no clear understanding of the construct ‘involvement’ from the perspective of children and young people with childhood‐onset disabilities.^2^, ^3^ Existing literature reviews, which were aimed at further understanding involvement or the experience of participation among children and young people with disabilities, explored (1) the distinction between the constructs ‘engagement’ and ‘involvement’ across health care, consumer and leisure research, economics, management, and psychological education;^9^ (2) the characterization of participation in literature related to the use of artificial intelligence in participation‐focused paediatric rehabilitation;^10^ (3) language use and definitions of participation in paediatric rehabilitation intervention studies incorporating participation outcomes;^11^ (4) the environmental impact on the participation of children and young people;^12^ and (5) the experience of participation of individuals with cerebral palsy when they transition into adulthood.^13^ However, these literature reviews either lack a specific focus on the voice of children and young people with childhood‐onset disabilities,^9^, ^10^, ^11^ or they focus primarily on the environmental impact on participation,^12^ or are limited to a specific age group or diagnosis.^13^ This limits a comprehensive understanding of the concept ‘involvement’ from the perspective of children and young people across different age groups and childhood‐onset disabilities. Without this deep understanding, we cannot fully measure involvement, define meaningful change, or assess its impact. Therefore, the aim of this scoping review was to gain a comprehensive understanding of the construct involvement in daily life activities from the perspective of children and young people with childhood‐onset disabilities.

## METHOD

### Study design

We conducted a scoping review of qualitative and mixed‐methods research studies. Scoping reviews are commonly used to gain an overview and understanding of the existing evidence and offer flexible methods for data analysis.^14^ Our scoping review was guided by the enhanced versions of Arksey and O'Malley's five steps^14^, ^15^, ^16^, ^17^ and was reported in accordance with the Preferred Reporting Items for Systematic reviews and Meta‐Analyses for Scoping Reviews (PRISMA‐ScR)^18^ guidelines. A review protocol was registered on the Open Science Framework.^19^

### Step 1: identifying the research question(s)

The research question guiding this scoping review was, ‘How do children and young people with disabilities describe their experience of involvement in daily life activities?’

Our published protocol included an additional question, related to whether conceptual ideas varied by context (i.e. country of children/young people, activity setting, social or individual context) and personal factors (i.e. child or young person age, type of disability, sex or gender); however, to allow a deeper examination of the core analytical question and maintain a cohesive and focused presentation of findings, this question is not addressed in this paper.

### Step 2: identifying relevant studies

We conducted a systematic literature search in February 2024 and a follow‐up search in February 2025 using PubMed, CINAHL and PsycINFO databases. We conducted pilot searches to define and refine search terms resulting in a final search string consisting of keywords, their synonyms, and thesaurus entries for (1) ‘child’ (‘child*’, ‘student*’, ‘youth’, ‘teen*’, ‘adolescen*’, ‘young adult’, ‘young adults’, pediatric*, paediatric*); (2) ‘disability’ (‘disab*’, ‘impair*’, ‘chronical medical condition’, ‘special needs’); (3) ‘participation involvement’ (‘involvement’, ‘participation’); (4) ‘qualitative research’ (‘qualitative’, ‘interview*’). The final search strings for each database are presented in Appendix S1. The search focused on documents published after 2001 (the year that the International Classification of Functioning, Disability and Health was published),^1^ without restrictions on language.^20^ To address the potential for missing relevant articles, we screened the reference lists of included articles as well as any literature review that was related to the current review topic.

### Step 3: study selection

After removing duplicates, the remaining articles were screened based on title and abstract, adhering to predefined inclusion and exclusion criteria (Table 1). For 25% of the articles, this was done independently by two reviewers with expertise on the participation construct (VCK and KQ) (92% agreement). The remaining articles were single screened by the same reviewers, with an additional 5% of randomly selected articles undergoing double screening (98% agreement). Articles meeting the inclusion criteria proceeded to the next screening stage, where the same reviewers screened their full text, applying the same inclusion and exclusion criteria. Articles published in languages other than English, German, or Dutch (i.e. the reviewers' proficient languages) were translated into English using the neural machine translation service DeepL (DeepL SE, Cologne, Germany) before full‐text screening. A total of 25% full‐texts were double‐screened by the same reviewers (96% agreement). The remaining full‐texts were single screened with 5% randomly selected articles undergoing double screening (86% agreement). Any discrepancies and uncertainties during the screening process were resolved through discussions among the reviewers and research team.

**TABLE 1:** Study inclusion and exclusion criteria.

Inclusion criteria	Exclusion criteria
Population	
Children and young people with disabilities, aged 0–24 years	No disability or diagnosed health conditions Studies involving individuals aged 25 years or older Studies involving stakeholders other than children and young people (e.g. caregivers, teachers), unless their role was only to support them, if needed (but not providing their own input)
Outcome	
The research objective focused on the experience of involvement, including the terms ‘involvement’, ‘participation’, and/or ‘engagement’	The research objective did not focus on the experience of involvement, participation, and/or engagement
Study designs	
Qualitative study designs Mixed‐methods study designs with a clear separation between qualitative and quantitative parts	Quantitative study designs Literature reviews, meta‐analyses Research in progress
Period of interest	
Articles published after 2001	Articles published before 2001
Article type	
Peer‐reviewed journal articles to ensure the inclusion of high‐quality research papers	Textbooks, textbook reviews Conference/workshop programmes Abstracts without additional information Non‐peer‐reviewed literature

### Step 4: charting the data

For data extraction, we developed data extraction documents which were pilot tested by two researchers (VCK and KQ) using five randomly selected articles and finalized through team discussions. Extracted data included publication year, country of origin, sample size, country of children/young people, child/young person age range and mean age, their diagnosis or type of disability, gender/sex, race/ethnicity, their family's socioeconomic background, and parental education. Additionally, we extracted verbatim narrative text at the subheading level from the results sections of the included studies, focusing on themes and categories describing children's and young people's experience of involvement in daily life activities. This included both researchers' descriptions and quotations from the child/young person. We defined child and young person involvement to also include the narrative representing the child or young person's voice of their experience of participation, because we defined involvement as the subjective dimension of participation.^2^ Narratives on participation attendance were excluded from the analyses, to maintain a focus on ‘involvement’, which represents a distinct dimension of participation according to the fPRC.^2^, ^3^

### Step 5: collating, summarizing, and reporting results

To gain an overview of the scope of research available, we used descriptive statistics to summarize the included studies according to their publication date, country of origin, sample size, and child/young person and family characteristics.

To gain a comprehensive understanding of the construct involvement, one researcher (VCK) conducted a thematic synthesis of the extracted narrative data using Taguette software.^21^ This process followed a three‐stage approach outlined by Thomas and Harden:^22^ (1) free line‐by‐line coding of the narrative data; (2) development of ‘descriptive themes’; and (3) creation of ‘analytical themes’. In the second stage, we remained close to the original text extracted from the included studies, summarizing content to develop descriptive themes. These were then interpreted and refined into analytical themes, guided by our research question. Throughout the process, the research team held regular discussions to review and refine both descriptive and analytical themes, helping to mitigate potential bias.

### Step 6: consultation

Two adults who identified as having a childhood‐onset disability were recruited through personal networks to provide feedback on our analytical themes, contributing to the refinement and interpretation of the results.^23^ The first author shared the preliminary themes and descriptive themes with them, after which they engaged in a structured discussion reviewing each theme and descriptive theme. Notes from this discussion were used to further refine the themes and descriptive themes in consultation with the author team.

The input of the two individuals with lived experience of childhood‐onset disability influenced revisions in the continuum of inner dedication or investment, initially phrased as in‐the‐moment thinking with four levels, which were subsequently renamed and developed into the five levels. In addition, the descriptive theme ‘experiencing the right amount of challenge’ was changed to ‘experiencing “active” participation’ to emphasize the active involvement rather than the presented challenge. The adjustments based on the consultation have been integrated into the findings and are not presented separately.

The individuals with a childhood‐onset disability who participated in this consultative process were compensated with an hourly payment for their time and insights.

## RESULTS

Our literature search retrieved 5241 documents, which, after removing duplicates (*n* = 1535), resulted in 3706 documents that were screened (Figure S1). After exclusions, 30 studies were included for this scoping review.

### Scope of the research

**FIGURE 1:**
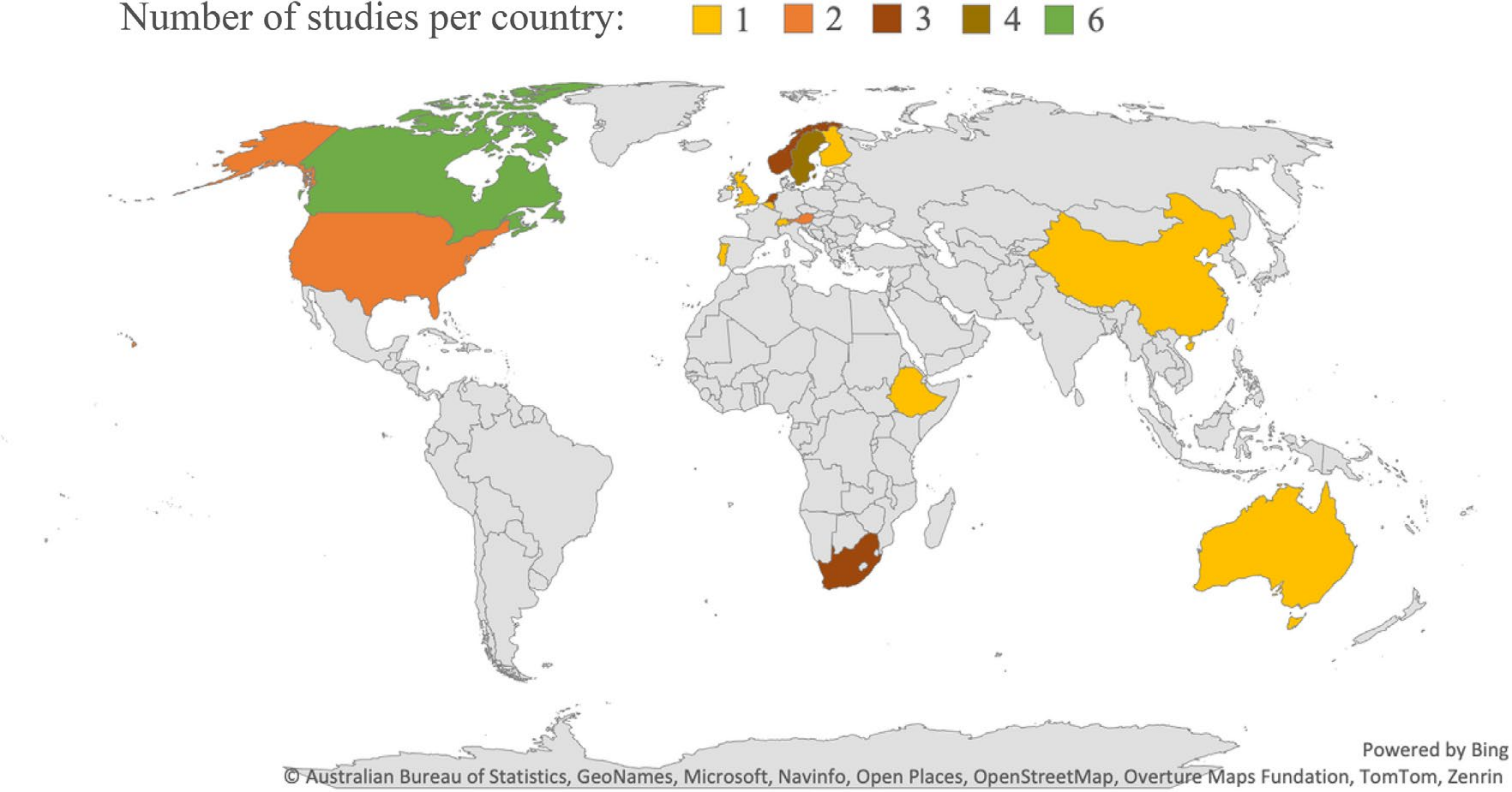
Countries represented in the included studies. Note: one study included children from both Sweden and South Africa.

The included studies were published between 2008 and 2024, with most of them (*n* = 19 out of 30) published after 2017. They were conducted in 15 different countries, with most in Canada (*n* = 6) (Figure 1). The sample size of the included studies ranged from 2 to 49 children and/or young people (mean = 15), with an age range of 5 years and 24 years across all included studies (Table 2).^4^, ^24^, ^25^, ^26^, ^27^, ^28^, ^29^, ^30^, ^31^, ^32^, ^33^, ^34^, ^35^, ^36^, ^37^, ^38^, ^39^, ^40^, ^41^, ^42^, ^43^, ^44^, ^45^, ^46^, ^47^, ^48^, ^49^, ^50^, ^51^, ^52^ Only data on the number of males and females were reported, with 25% to 100% being male. A broad range of disabilities were represented in the included studies, with physical disability being the most prevalent (Table 2). Only one included study reported on the family's socio‐economic background^24^ and parental education.^25^

**TABLE 2:** Included studies.

Study (country)	Sample (*n*); sex (% male)	Mean child/young person age (SD); range (years)	Child/young person diagnosis/type of disability	Child/person race/ethnicity	Family socioeconomic status or parental education
Asbjørnslett et al.^34^ (Norway)	14; 57%	15.1 (1.7); (13–18)	PD	NR	NR
Asbjørnslett et al.^38^ (Norway)	15; 60%	NR (NR); (12–14)	BVI, PD	NR	NR
Bantjes et al.^32^ (South Africa)	15; NR	NR (NR); (12–18)	CP	White, Coloured, African	NR
Brunes et al.^42^ (Norway)	2; 50%	17.5 (5.5); (12–23)	BVI	NR	NR
Byhlin et al.^29^ (Sweden)	14; 29%	NR (NR); (21–23)	ID	NR	NR
Conchar et al.^27^ (South Africa)	15; NR	NR (NR); (12–18)	CP	White, Coloured, African	NR
Correia et al.^37^ (China)	5; 60%	16.2 (2.1); (13–19)	Other (dyslexia)	Chinese	NR
Coussens et al.^24^ (Belgium)	16; NR	6.8 (0.9); (5.4–9)	ASD, PD, multiple, Other	NR	Middle class
Dahan‐Oliel et al.^43^ (Canada)	11; 45%	17.0 (1.6); (13.9–19.4)	BVI, PD	NR	NR
Devine^35^ (USA)	16; 44%	20.6 (2.1); (18–24)	BVI, CHC, CP, PD, SB	Asian American	NR
Gantschnig et al.^26^ (Austria)	5; 60%	9.4 (1.5); (8–12)	CP, ID, PD, multiple	NR	NR
Harding et al.^44^ (Canada)	6; 83%	11.3 (1.9); (8–13)	CHC, PD	Caucasian	NR
Harries et al.^45^ (UK)	25; 48%	NR (NR); (14–18)	CHC	NR	NR
Huus et al.^41^ (Sweden/South Africa)	49; NR	NR (NR); (7–18)	ID	NR	NR
Knibbe et al.^46^ (Canada)	11; 36%	NR (NR); (12–18)	CP, PD	NR	NR
Kreider et al.^47^ (USA)	19; 84%	13.9 (1.2); (11–16)	Other (ADHD)	NR	NR
Krieger et al.^25^ (Switzerland)	6; 100%	17.3 (2.0); (15–20)	ASD	NR	Low, middle, high education
Lauruschkus et al.^48^ (Sweden)	16; 44%	9.1 (1.1); (8–11)	ID	NR	NR
Meier et al.^49^ (Austria)	19; 37%	15.7 (1.7); (14–20)	BVI	NR	NR
Nap‐ van der Vlist et al.^33^ (the Netherlands)	31; 39%	NR (NR); (8–18)	CHC	NR	NR
Pereira et al.^31^ (Portugal)	14; 50%	9.8 (1.1); (8–11)	BVI, CP, ID, PD, other (retinopathy, Duchenne muscular dystrophy)	NR	NR
Pontes et al.^50^ (Canada)	5; 60%	10.2 (1.6); (7–11)	PD	NR	NR
Renwick et al.^40^ (Canada)	24; 63%	NR (NR); (13–24)	ASD, CP, ID, Other (Down syndrome, fetal alcohol spectrum disorder, Prader–Willi syndrome)	NR	NR
Simpson et al.^4^ (Australia)	4; 50%	11.6 (0.6); (10.7–12.1)	ASD	NR	NR
Ullenhag et al.^28^ (Sweden)	14; 71%	17.3 (3.2); (14–24)	CP	NR	NR
Vänskä et al.^30^ (Finland)	9; 56%	7.2 (1.8); (5–10)	PD	NR	NR
Veldhorst et al.^39^ (the Netherlands)	25; 52%	13.5 (1.1); (11–15)	BVI, PD	NR	NR
Volfson et al.^51^ (Canada)	9; 33%	15.9 (1.0); (14–17)	SB	Caucasian, Armenian, Indian, Egyptian, Filipino	NR
Wintels et al.^36^ (the Netherlands)	23; 57%	15.3 (2.3); (12–17)	CP	NR	NR
Yeshanew et al.^52^ (Ethiopia)	17; 29%	19.9 (NR); (17–22)	BVI	NR	NR

*Note*: Ages are presented in decimal years as reported in the original studies. Race/ethnicity information is as presented in the original studies.

Abbreviations: ADHD, attention‐deficit/hyperactivity disorder; ASD, autism spectrum disorder; BVI, blind or visually impaired; CHC, chronic health condition (e.g. asthma, diabetes); CP, cerebral palsy; ID, intellectual disability; multiple, multiple disabilities; PD, physical disability; SB, spina bifida; Other, other disabilities; NR, not reported.

### Concepts describing involvement

**FIGURE 2:**
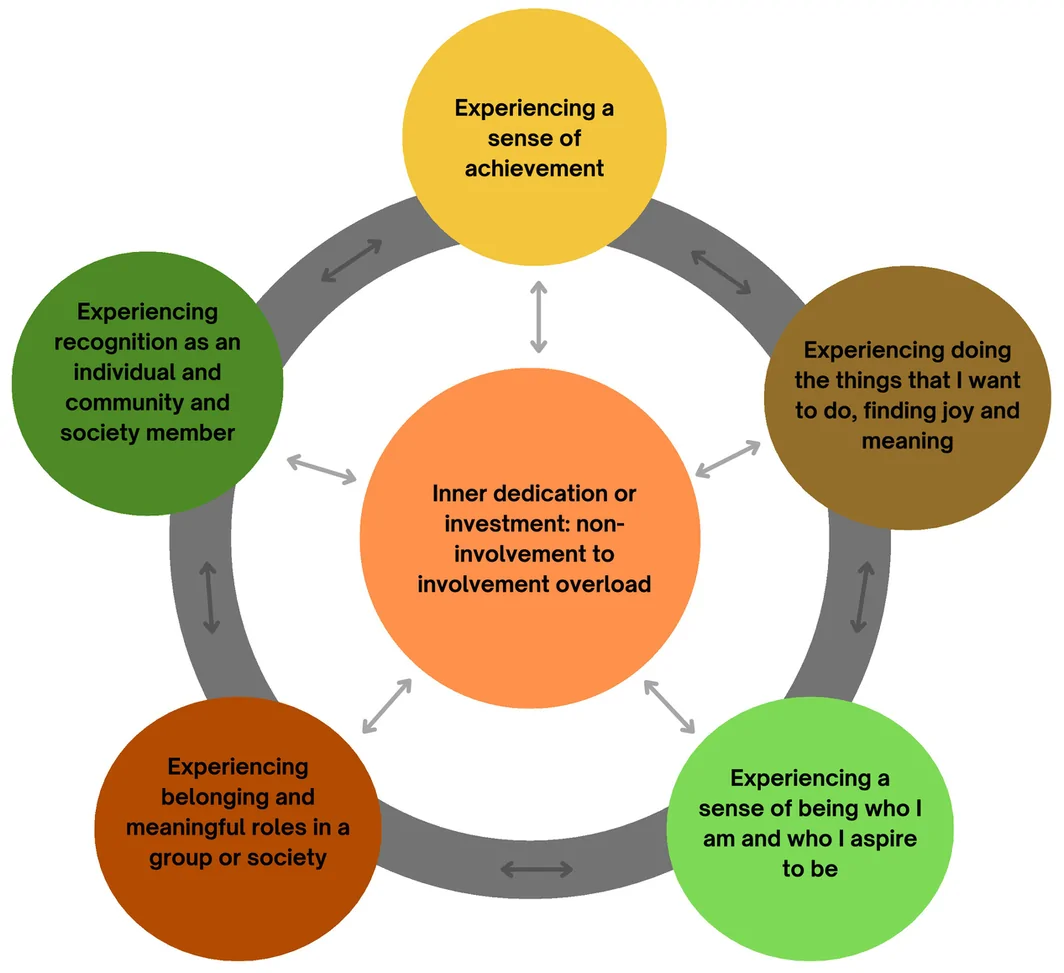
Conceptual ideas related to the experience of involvement.

The thematic synthesis revealed six analytical and descriptive themes that represent conceptual insights into the experience of ‘involvement in activities’ from the perspective of children and young people with childhood‐onset disabilities: (1) a continuum of inner dedication or investment, ranging from non‐involvement to involvement overload; (2) experiencing a sense of achievement; (3) experiencing doing the things that I want to do, finding joy and meaning; (4) experiencing a sense of being who I am and who I aspire to be; (5) experiencing belonging and meaningful roles within a group or society; and (6) experiencing recognition as an individual and as a member of the community and society. The continuum of inner dedication or investment in an activity focus and dedication to the activity (theme/concept 1) can be understood as the in‐the‐moment experience of involvement, representing the core of the involvement experience (Figure 2). Themes/concepts 2 to 6 represent how children and young people process and reflect on their involvement experience during or after attending an activity, and/or doing one, displayed in a circle surrounding theme 1.

### Continuum of inner dedication or investment: non‐involvement to involvement overload

**FIGURE 3:**
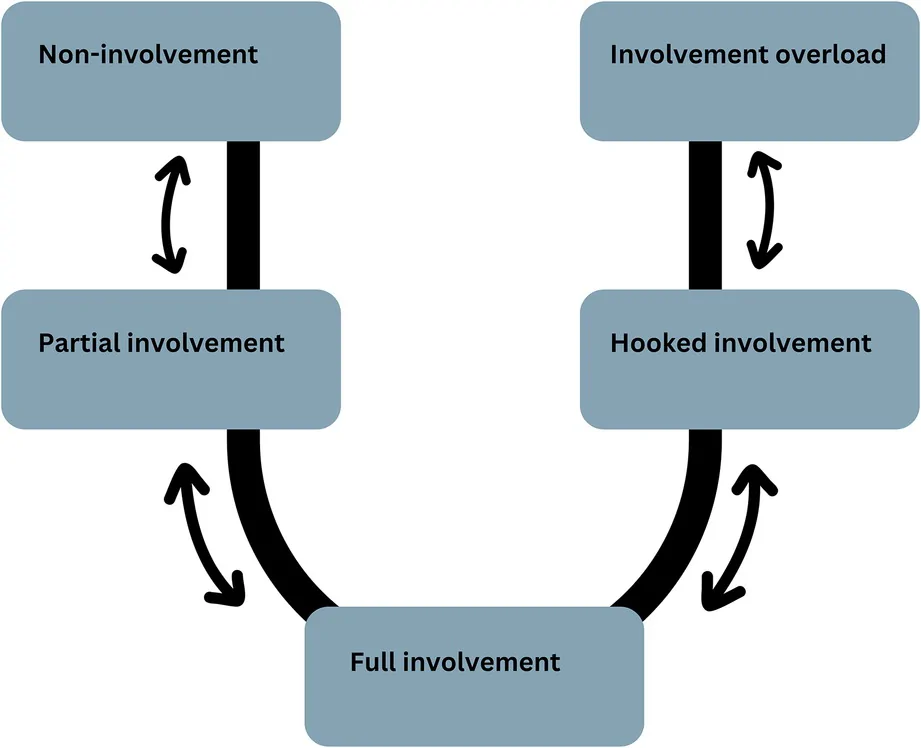
Continuum of levels of involvement.

Children and young people described their experience of involvement in activities as the amount of inner dedication to or investment in an activity. This was reflected in children and young people's experience of in‐the‐moment mental presence or the mental energy they devoted to the activity while attending and/or doing it, which was described by Gantschnig et al.^26^ as entailing children and young people ‘actively participating with their mind’. Analyses of these data resulted in a continuum of five levels forming a U‐shape (Figure 3) and representing the following descriptive themes: (1) lack of inner dedication or investment in an activity (non‐involvement); (2) mentally shifting in and out of an activity (partial involvement); (3) full inner dedication or investment in an activity (full involvement); (4) being hooked to an activity (hooked involvement); and (5) exhaustion through involvement (involvement overload).

#### Lack of inner dedication or investment in an activity (non‐involvement)

This first level on the continuum of involvement represented non‐involvement, a state that children and young people described as their mind not being actively engaged with the activity they were attending and/or doing. This was illustrated by a child/young person in Simpson et al.^4^ who said about activities where she was not involved, ‘I get distracted very easily’. Similarly, a child/young person in Conchar et al.^27^ shared an experience of non‐involvement: ‘I just sit here for that period’. Experiencing non‐involvement through a lack of in‐the‐moment dedication or inner investment in an activity was also described with feelings of restlessness.^25^

#### Mentally shifting in and out of an activity (partial involvement)

This second level on the continuum of involvement represented partial involvement, which was described by children and young people with childhood‐onset disabilities as having the mind shift in and out of the activity. For example, a child/young person in Simpson et al.^4^ described being ‘somewhat involved’ as ‘[letting] my mind stray a little and think of other things’. In contrast, ‘full involvement’ in an activity was depicted as having the mind focused on that activity such as ‘when making truffles with her family, the conversation was on the task “instead of like ‘oh how are you doing?’ it's more of a ‘okay so I'll roll three more truffles'”’.^4^ Similarly, another child/young person in Simpson et al.^4^ illustrated how ‘her focus of involvement could move back and forth between concurrent activities’. She said, “Sometimes I'd just do the puzzle and sort of ignore the music, and sometimes I'd sort of dance to the music and stop the puzzle”’.

#### Full inner focus and dedication to an activity (full involvement)

The third level of involvement entailed being fully emersed in an activity, which was described as a full dedication to an activity without getting distracted by anything around them. Children and young people described it as ‘I just let go [of everything around me]’,^27^ ‘I got really into it then’,^28^ and ‘having ‘a very small amount of thoughts' and […] when ‘that's the only thing that I think while I'm doing that’.^4^ This was described as being ‘irrespective of [their] enjoyment of the activity’,^4^ meaning that full inner focus and dedication could occur in pleasant as well as unpleasant activities (e.g. peer conflicts). In the case of pleasant activities, experiencing involvement through this deep inner dedication or investment in the activity was described as being associated with feelings of relaxation and calm; this was expressed, for example, by a child/young person in Simpson et al:^4^ ‘The overall feel, the relaxation, […]’. Additionally, such experiences were described as feelings of sudden cheerfulness as expressed by a child/young person in Conchar et al:^27^ ‘Your body goes into jitters, because there are butterflies because you are actually on the pitch’.

#### Being hooked to an activity (hooked involvement)

The fourth level of involvement entailed an experience of being hooked to an activity and represents a more extreme version of full inner dedication or investment. Children and young people with childhood‐onset disabilities described this experience as not being able to stop with the activity, which some perceived as positive and others as negative. The positive aspect of being hooked to the activity was expressed by a child/young person in the study by Gantschnig et al.,^26^ who stated, ‘I like doing it so much that I do not want to stop’. Conversely, the negative aspect of being hooked to the activity was characterized as an addictive behaviour. For instance, Simpson et al.^4^ described involvement in video games as addictive, stating,

He described the games as addictive, ‘I've put 68 hours into it, so you can see I'm pretty … hooked,’ and playing the games was not always calming: this depended on the type of game he played. For example, as he described the experience: ‘It's like you hate it and you really just want to stop playing … basically really addicting because you do just want to complete the level, and you keep on playing and raging and raging’.

#### Exhaustion through involvement (involvement overload)

The fifth level of involvement encompassed experiences of too much involvement or involvement overload, which were described as exhausting. For example, a young person with autism spectrum disorder in Krieger et al.^25^ expressed involvement overload by a desire for reduced involvement in social situations and developing strategies to manage their involvement:

It is a tension. On one hand, it is nice to get an invitation and to be together with people that I already know a bit. On the other hand, it is exhausting to go home late. … I have to learn to use less energy to hang out with others. My girlfriend has helped me a lot to this end. But yes, it is exhausting.

Similarly, children and young people in the study by Byhlin et al.^29^ reported opting out of events because of overload, with one child/young person noting, ‘then I can say I want to participate but I can't because it's too much’.

### Experiencing a sense of achievement

In children and young people's reflections about their involvement, they described how involvement can be experienced as a sense of achievement. This was described in two ways, representing the following descriptive themes: (1) experiencing a sense of competence; and (2) experiencing ‘active’ participation.

#### Experiencing a sense of competence

Children and young people described involvement as experiencing a sense of competence such as through achievement. This encompassed the children and young people's own perspective of being good at something; examples included experiencing mastering scholarly activities^26^ and winning games.^27^, ^30^ For example, children and young people stated, ‘We were swimming like dogs do, and I was always first’^26^ or ‘the game I like the most is when we have to pretend that we're statues and we can't move. If the observer sees someone moving, this child is out and changes place with the observer. The game is really good fun and I could play it forever because I'm very good at it!’^31^

#### Experiencing ‘active’ participation

Children and young people with childhood‐onset disabilities reported experiencing a lack of involvement when activities were adapted to such an extent that they felt bored and unchallenged. Some indicated that merely observing others participating did not yield a sense of involvement. For them, experiencing involvement entailed active participation in the activity. Bantjes et al.^32^ illustrated this by noting,

Participants described their experience of being excluded from active participation and cast into the role of spectator, which they experience as boring and demotivating. They expressly wish to be actively involved in sport and to take part alongside their peers; for them the definition of participation does not include being a spectator.

This was illustrated by a child/young person sharing, ‘It's like boring after a few years of cheering your team on and that. But it's exciting for the younger kids when it's like their first or their second time of doing it. So after about four or five times you cheer a bit, but you mostly sit there and watch’.^32^

### Experiencing doing the things that I want to do, finding joy and meaning

Children and young people with childhood‐onset disabilities described involvement in activities as the experience of doing what they want, in the way they want. This analytical theme comprises three descriptive themes: (1) experiencing doing what I want; (2) experiencing the right balance of space and support; and (3) experiencing feelings of enjoyment, excitement, and meaningfulness.

#### Experiencing doing what I want

Children and young people with childhood‐onset disabilities described involvement as experiencing doing what they want to do and the way they want to do it. For example, Byhlin et al.^29^ ‘described participation in terms of their own will […]’, and Nap‐Van Der Vlist et al.^33^ as ‘participating in his own way’. This included experiencing having influence on their own involvement in activities, as illustrated by the quote ‘that I can decide what I want to do. I can play, and do tricks and go down the waterslide’.^30^

#### Experiencing the right balance of space and support

Children and young people with childhood‐onset disabilities also described the experience of involvement as finding the right balance between having space and receiving support. For some children and young people with childhood‐onset disabilities, involvement in activities felt compromised when too much support was provided by assistants, as illustrated by Asbjørnslett et al.:^34^ ‘the feeling of being a student among others could be disrupted by having an assistant who was over‐helpful, telling them how to do the group work or giving hints during tests’. Similarly, the involvement was also experienced as receiving trust from adults and having the ability to take risks, as one child/young person in Devine et al.^35^ noted, ‘it was like I was in a little bubble, and if I stepped out they were afraid I'd get hurt’.

#### Experience feelings of enjoyment, excitement, and meaningfulness

Children and young people with childhood‐onset disabilities described involvement as feelings of enjoyment, fun, and meaningfulness. These feelings were also linked to sensory and motor aspects of an activity. For example, Vänskä et al.^30^ stated, ‘meaningful participation involved memorable experiences and a feeling of excitement, such as daring to swing high, running fast when playing tag, or cycling at speed’. Children and young people also expressed these emotions in their own words: ‘The excitement! This thing (gestures to his legs) could be shaking and I should stop it, that's exciting’^32^ or ‘a big burst of enjoyment’.^4^ In some cases, involvement in activities was even described as the meaning of life: ‘I should say that participation is the meaning of life’.^28^

### Experiencing a sense of being who I am and who I aspire to be

Children and young people with childhood‐onset disabilities described involvement in activities as experiencing a sense of being who I am and who I aspire to be. This resulted in two descriptive themes: (1) the sense of being my authentic self; and (2) the sense of becoming the person I aspire to be.

#### The sense of being my authentic self

Children and young people with childhood‐onset disabilities described involvement as being able to be who they are. In other words, as one child/young person in the study by Conchar et al.^27^ shared, ‘I can be myself; express myself; do different things with my body’. Often, experiencing involvement through a sense of being their authentic self was experienced within their families. For example, a child/young person in Nap‐Van Der Vlist et al.^33^ noted, ‘It also had an impact at home, because there I could really be who I wanted to be. When I came home from a long day, my mom was the one noticing that it did not go well’. Similar experiences were echoed in interactions with friends, where involvement meant being accepted regardless of their differences. One child/young person in Wintels et al.^36^ remarked that involvement ‘means they [friends] don't mind if I sometimes, that I'm dreaming or making odd movements, or that I have trouble at gym or things like that. Yeah, they just help me and then, yeah, …they do accept me the way I am’.

#### The sense of becoming the person I aspire to be

Children and young people with childhood‐onset disabilities described involvement as experiencing a process of developing into the person they aspired to be or, as articulated by Correia et al.,^37^ ‘whom they wished to become’. A child/young person in the study by Ullenhag et al.^28^ expressed this notion as ‘being able to ‘realise oneself […]’ This process was particularly emphasized in the context of transitioning from childhood to adulthood, where ‘participation was expressed to be an opportunity to find your own way in life’.^28^

### Experiencing belonging and meaningful roles in a group or society

Involvement in activities was described by children and young people with childhood‐onset disabilities as experiencing belonging and having meaningful roles in a group or society. This was illustrated through the three descriptive themes: (1) experiencing belonging; (2) an experience of social connection; and (3) an experience of having a meaningful role in an activity, group, and/or society.

#### Experiencing belonging

One way of experiencing involvement, as described in the included studies, was the experience of belonging. Gantschnig et al.^26^ noted, ‘the other feature of involvement is the experience of belonging while doing things together with classmates, for example, singing a song or cooking a meal’. Similarly, other authors highlighted that involvement ‘felt for them [children and young people with childhood‐onset disabilities] as “being part” or “belonging to”’^24^ and was experienced as ‘inclusion and belonging’.^28^ For some children and young people with childhood‐onset disabilities, experiencing involvement meant ‘to be where things happen’,^26^, ^34^ regardless of whether they were actively doing what others were doing. This was, for example, illustrated by a child/young person's reflection in Gantschnig et al.^26^: ‘I was outside earlier today, sitting on a bench. I could not play, but at least I could see what they were playing. They played cops and robbers. It was so funny!’

Additionally, children and young people described involvement as an experience of belonging through the types of activity they were involved in. Attending and/or doing socially and/or culturally valued activities, such as those perceived as ‘cool’, provided a sense of involvement. For example, Asbjørnslett et al.^38^ remarked ‘[a] 13‐year‐old girl stated: “Everybody is on Facebook,” adding that her “friends” went on Facebook every day to keep up with each other’.

#### Experience of social connection

Experiencing involvement was also described as experiencing a ‘connection to their social environment’.^39^ Vänskä et al.,^30^ for example, stated that the ‘main elements of meaningful participation are: […] social involvement’. This includes experiencing ‘“friendship” and “companionship”’.^24^ A child/young person in Bantjes et al.^32^ and Conchar et al.^27^ described it as:

There's also a nice family feel, like everybody supports you there. … Basically, like if I finish the race, the marshals and that, they clap and say well done, even though I haven't done such a great time. …Yes, you can always cheer with your team mates and that. It's like a nice group feel when they're cheering you on.

Similarly, a child/young person in Renwick et al.^40^ described that social connection was a way of experiencing involvement: ‘what makes me feel a part of my community is the people around me and the way they make me feel good. They talk to me, which makes me feel like I'm a part of the community and sometimes do things for me like get me stuff that they know that I like, they buy me stuff, that sort of thing’.

#### Experience having a meaningful role in an activity, group, or society

Children and young people with a childhood‐onset disability described their experience of involvement as having a meaningful role within an activity, group, community, and society. This included their sense of contributing to these groups and activities as a community and society member. For example, a child/young person in Renwick et al.^40^ experienced involvement through ‘being a member of a community; and contributing to a community through meaningful roles’. Another child/young person in Devine et al.^35^ noted, ‘I am a member of this team, plain and simple; no more or less […]’ Similarly, a child/young person in Ullenhag et al.^28^ stated that involvement means ‘that I can be part of the community, and contribute, to help others, that I can contribute with my resources and my knowledge, that I am allowed to be a resource for society’.

### Experiencing recognition as an individual and community and society member

Children and young people with childhood‐onset disabilities described involvement as experiencing recognition both as individuals and as members of their community and society. This analytical theme consists of two descriptive themes: (1) experiencing being noticed by others, but not stared at; and (2) experiencing being appreciated and understood by others.

#### Experiencing being noticed by others, but not stared at

Children and young people with childhood‐onset disabilities reported that involvement meant for them being noticed or seen by others, as opposed ‘to the experience of feeling invisible’.^32^ Huus et al.^41^ highlighted the difference between being noticed and being ignored, noting that ‘the children felt ignored as they stated that “some people don't listen”’. Being noticed by others also extended to ensuring that activities and the equipment were accessible. For example, Bantjes et al.^32^ stated, ‘They articulate a wish for greater recognition of sport for persons with disabilities’ with a child/young person expressing, ‘I feel that in South Africa they notice normal people—and that's it. They don't really see there's another type of person that's disabled […]’.^32^ However, unwanted attention such as by being stared at resulted in the experience of non‐involvement. One child/young person in Devine et al.^35^ shared:

When I'd roll into the pool, he'd either jump off the guard stand or run out of the office to meet me and fist‐pumped me saying ‘here's my swimming super star!’ Then as I'd complete a lap, he'd cheer really loud. Finally, I said to him ‘Ah, thanks for helping me in and out of the pool with the lift and everything bud, but I'm not swimming across Lake Erie dude. I'm just swimming laps’. I just wanted to tone him down.

#### Experiencing being appreciated and understood by others

Children and young people with childhood‐onset disabilities described involvement as experiencing being appreciated and understood by others. This included being viewed as an enrichment, rather than a burden, when attending and/or doing an activity, ‘leading to recognition for who they are’.^24^ Often, included articles talked about non‐involvement as a lack of experiencing appreciation, fear of embarrassment, and being judged for who they are. This is illustrated by Gantschnig et al.^26^ noting, the ‘experience of not being one of the favorite children, as a feature of not feeling involved’. One child/young person described the frustration of not being chosen for team games resulting in experiencing non‐involvement: ‘If we play a game in physical education, two children have to choose their members. Yes, and I am always the last one to be chosen. I have to wait and wait. It is so annoying. I can't stand it’. Similarly, another child/young person expressed a desire for acceptance: ‘Well I want to be accepted, I want people to […] not judge me by the way I look, the way I act, […]. I want to feel important and to be noticed’.^40^

## DISCUSSION

Participation in daily life activities is recognized as a key aspect of child and young person health and well‐being,^1^, ^53^ social inclusion,^53^ and skill development.^54^ In the fPRC framework,^2^ participation comprises two dimensions: attendance and involvement. While ‘attendance’ is well‐defined, the notion of ‘involvement’ has remained conceptually diffuse. This scoping review synthesized evidence from the past two decades to enhance the conceptual clarity about ‘involvement’ from the perspective of children and young people with childhood‐onset disabilities.

Our review identified six key concepts that characterize how children and young people experience involvement in daily life activities. The first concept emphasizes a continuum of inner dedication or investment, reflecting the immediate, in‐the‐moment experience that defines involvement. This understanding aligns with previous research, which suggests that involvement is closely linked to whether children and young people are thinking about the same thing as they are doing.^55^ In other words, ‘when people have their mind on what they are doing they perceive higher involvement than if they are only doing, without thinking about what they are doing’.^55^ This perspective of involvement partly overlaps with flow theory,^56^ which describes flow as ‘the state in which people are so involved in an activity that nothing else seems to matter; the experience itself is so enjoyable that people will do it even at great cost, for the sheer sake of doing it’.^56^ In our review, children and young people with childhood‐onset disabilities described similar experiences, which are captured under ‘full involvement’ and partly ‘hooked involvement’. However, our findings extend beyond flow by demonstrating that the experience of involvement can occur in both pleasant and unpleasant activities (e.g. peer conflicts). This is supported by recent research showing that full involvement in physical activities does not necessarily mean young people are enjoying the activity.^57^ Most studies included in our scoping review, however, focused on pleasant activities, introducing a positivity bias that may have shaped our results. To mitigate this, future research should also explore children and young people's experiences of involvement in unpleasant or undesirable activities.

Our findings identified five levels of involvement including non‐involvement, partial involvement, full involvement, hooked involvement, and involvement overload. Viewing inner dedication and investment as a continuum is consistent with recent research exploring experiences of involvement in physical activities from the perspective of young people with CP and their parents.^57^ In this previous research, the continuum of involvement ranged from helpful to hindering levels of involvement. Helpful involvement was characterized as a ‘high level of effort, concentration, readiness, and thought’,^57^ whereas hindering involvement referred to ‘too much’ involvement, leading to negative experiences such as frustration, exhaustion, and displeasure. Findings from this scoping review further refine this continuum by categorizing states of inner dedication and investment into five levels. Future research should seek to validate or refine these levels from the perspective of children and young people with childhood‐onset disabilities, which could also further inform measurement of children and young people's involvement in daily life activities.

Beyond the involvement levels, findings of this review revealed five additional concepts of children and young people's involvement, reflecting how children and young people process, interpret and reflect on their experiences of involvement. These include children and young people's experience of sense of achievement, doing the things that they want to do, finding joy and meaning, sense of being who they are and who they aspire to be, belonging and meaningful roles within a group or society, and recognition as an individual and as a member of the community and society. These experiences may align with components in the fPRC framework;^2^ specifically, the dynamic transactional processes that describe hypothesized reciprocal relationships between participation (i.e. attendance, involvement) and participation‐related constructs (i.e. the child's environment/context, activity competencies, sense of self, and preferences).^2^, ^3^ These processes are hypothesized to influence one another, shaping both participation experience and participation‐related constructs.

Two of the concepts identified in our results, ‘experiencing belonging and meaningful roles within a group or society’ and ‘sense of being who I am and who I aspire to be’, align closely with the transactional relationship between participation and a child or young person's sense of self in the fPRC. This relationship is characterized by children and young people's self‐perception (e.g. autonomy, optimism, self‐determination, and self‐esteem) and their engagement in present and future activities,^2^ jointly influencing both the child/young person's participation experiences and their sense of self. These two conceptual ideas of involvement also resonate with self‐determination theory,^58^ particularly the experience of relatedness (i.e. feeling connected to, valued by, and significant to others), which is conceptually linked to the sense of self construct in the fPRC.

Similarly, the conceptual idea ‘experiencing doing the things that I want to do, finding joy and meaning’ aligns with the hypothesized relationship between participation and a child or young person's preferences, characterized by the ability and opportunity to choose and the necessity to comply with activities that must be done.^2^ The conceptual idea ‘sense of achievement’ maps onto the interaction between activity competence and participation, described in the fPRC framework as acting and learning, which mutually influence participation experiences and the development of activity competencies.

Finally, ‘experiencing recognition as an individual and community and society member’ aligns with the transactional relationship between the environment/context and participation. In the fPRC framework,^2^ this relationship reflects the provision and regulation of participation opportunities, with, again, reciprocal influences on both the participation experience and the environment/context.

The theoretical alignments of the concepts resulting from this study with the fPRC's dynamic transactional processes between participation and participation‐related constructs may contribute to a more nuanced understanding of child and young person experience of involvement and relationships with related constructs; however, it also raises questions about conceptual overlaps among them and, thus, how to comprehensively measure and intervene on involvement. Research is underway to explore the relationship among the fPRC concepts through factor analysis which may provide further guidance on the involvement concept and its relationship to the dynamic processes. Such knowledge may help determine whether involvement encompasses all six concepts revealed in this study and if these represent distinct, separate concepts. This distinction will be crucial for further developing involvement‐focused measurement and intervention.

The findings of this scoping review need to be interpreted while considering several limitations. Most of these limitations relate to what data were available to inform the review analyses, and the impact this might have on the findings. First, while included studies were all focused on participation, they each asked different questions about child and young person involvement. Our findings may have differed if the included studies were more homogenous in this regard. Third, some studies included quotes that were translated into English, which may have resulted in loss or misinterpretation of meaning during analysis. This might be especially important if the experience of involvement (as conceptualized) is culturally influenced. Fourth, most studies included in this review reported on male and female sex or gender, limiting the ability to represent gender diversity more inclusively. Fifth, severity of disability was not reported, which might be a more informative factor than diagnostic categories in understanding involvement. Sixth, only 25% of the retrieved articles and 5% of randomly selected articles were double screened on title/abstract and full‐text levels. Although interrater agreement across the double‐screening processes was generally high (i.e. 86%–98%), this approach may have introduced study selection bias. Seventh, while we included adults with lived experience of childhood‐onset disabilities to provide feedback on our analytical themes, contributing to the refinement and interpretation of the results, involving them throughout the process of the review would have further increased the quality of our work.^23^ Eighth, our protocol included a research question exploring whether the involvement construct was influenced by contextual or personal factors. We were unable to thoroughly address this question in this article, leaving it as an open question for future research.

## CONCLUSION

There is growing recognition of the importance of involvement as a dimension of child and young person participation, as evidenced by an increasing body of literature examining involvement among children and young people with childhood‐onset disabilities across diverse countries and settings. This scoping review identified six concepts of involvement. The first represents a continuum of inner dedication or investment to an activity, characterized by five levels. The remaining five concepts capture how children and young people process, interpret, and reflect on their involvement experiences. Future research should focus on examining child and young person involvement in unpleasant or mandated activities, validating or refining the identified five levels of involvement, and exploring the role of conceptual ideas in relation to related concepts and terms in developing interventions to enhance involvement.

## CONFLICT OF INTEREST STATEMENT

The authors have stated that they had no interests that might be perceived as posing a conflict or bias.

## Supporting information


**Appendix S1:** Sample search string.

**Figure S1:** Study selection.

## Data Availability

Data sharing not applicable ‐ no new data generated, or the article describes entirely theoretical research.

## References

[1] World Health Organization . International Classification of Functioning, Disability and Health. Geneva: World Health Organization; 2001.

[2] Imms C , Granlund M , Wilson PH , Steenbergen B , Rosenbaum PL , Gordon AM . Participation, both a means and an end: a conceptual analysis of processes and outcomes in childhood disability. Dev Med Child Neurol 2017; 59: 16–25. 27640996 10.1111/dmcn.13237

[3] Imms C. The nature of participation. In: Imms C , Green D , editors. Participation: Optimising Outcomes in Childhood‐Onset Neurodisability. London: Mac Keith Press; 2020: 5–11.

[4] Simpson K , Imms C , Keen D . The experience of participation: eliciting the views of children on the autism spectrum. Disabil Rehabil 2022; 44: 1700–8. 33771078 10.1080/09638288.2021.1903100

[5] Granlund M , Imms C . Participation as a means ‐ implications for intervention reasoning. Front Rehabil Sci 2024; 5: 1–13. 10.3389/fresc.2024.1399818 PMC11236800 38994330

[6] Hanzen G , van Nispen RMA , van der Putten AAJ , Waninge A . Participation of adults with visual and severe or profound intellectual disabilities: Definition and operationalization. Res Dev Disabil 2017; 61: 95–107. 28064027 10.1016/j.ridd.2016.12.017

[7] Martin Ginis KA , Sinden AR , Bonaccio S , et al. Experiential Aspects of Participation in Employment and Mobility for Adults With Physical Disabilities: Testing Cross‐Sectional Models of Contextual Influences and Well‐Being Outcomes. Arch Phys Med Rehabil 2024; 105: 303–313. 37607656 10.1016/j.apmr.2023.08.004

[8] Adair B , Ullenhag A , Rosenbaum P , Granlund M , Keen D , Imms C . Measures used to quantify participation in childhood disability and their alignment with the family of participation‐related constructs: a systematic review. Dev Med Child Neurol 2018; 60: 1101–16. 30022476 10.1111/dmcn.13959

[9] Steinhardt F , Dolva AS , Jahnsen R , Ullenhag A . Exploring two subdimensions of participation, involvement and engagement: a scoping review. Scand J Occup Ther 2022; 29: 1–23. 34242105 10.1080/11038128.2021.1950207

[10] Kaelin VC , Valizadeh M , Salgado Z , Sim JG , Anaby D , Boyd AD , et al. Capturing and operationalizing participation in pediatric re/habilitation research using artificial intelligence: a scoping review. Front Rehabil Sci 2022; 3: 1–14. 10.3389/fresc.2022.855240 PMC9340801 35919375

[11] Imms C , Adair B , Keen D , Ullenhag A , Rosenbaum P , Granlund M . ‘Participation’: a systematic review of language, definitions, and constructs used in intervention research with children with disabilities. Dev Med Child Neurol 2015; 58: 29–38. 26411643 10.1111/dmcn.12932

[12] Kramer JM , Olsen S , Mermelstein M , Balcells A , Liljenquist K . Youth with disabilities' perspectives of the environment and participation: a qualitative meta‐synthesis. Child Care Health Dev 2012; 38: 763–77. 22372695 10.1111/j.1365-2214.2012.01365.x

[13] Cleary SL , Morgan PE , Wallen M , Honan I , Shields N , Munzel FE , et al. Experiences of participation in daily life of adolescents and young adults with cerebral palsy: a scoping review. Dev Med Child Neurol 2025; 67: 572–590. 39673293 10.1111/dmcn.16196

[14] Peters MDJ , Marnie C , Tricco AC , Pollock D , Munn Z , Alexander L , et al. Updated methodological guidance for the conduct of scoping reviews. JBI Evid Synth 2020; 18: 2119–26. 33038124 10.11124/JBIES-20-00167

[15] Arksey H , O'Malley L . Scoping studies: Towards a methodological framework. Int J Soc Res Methodol 2005; 8: 19–32.

[16] Peters MDJ , Godfrey C , McInerney P , Munn Z , Tricco AC , Khalil H . Chapter 11: scoping reviews. In: Aromataris E , Munn Z , editors. JBI Manual for Evidence Synthesis. The Joanna Briggs Institute; 2020.

[17] Levac D , Colquhoun H , O'brien KK . Scoping Studies: advancing the Methodology. Implement Sci 2010; 5: 1–9. 20854677 10.1186/1748-5908-5-69 PMC2954944

[18] Tricco AC , Lillie E , Zarin W , O'Brien KK , Colquhoun H , Levac D , et al. PRISMA Extension for scoping reviews (PRISMA‐ScR): checklist and explanation. Ann Intern Med 2018; 169: 467. 30178033 10.7326/M18-0850

[19] Kaelin V , Quaaden K , Newman‐Griffis D , Lindgren H , Granlund M , Imms C . Towards a better understanding of Involvement in daily life activities from the perspectives of children and youth: protocol of a scoping review [Internet]. OSF; 2024 Mar [cited 2025 April 21]. 10.17605/OSF.IO/HNVBJ

[20] Dan B. The many languages of developmental disability research. Dev Med Child Neurol 2022; 64:808–809. 35642089 10.1111/dmcn.15231

[21] Rampin R , Rampin V . Taguette: open‐source qualitative data analysis. J Open Source Softw 2021; 6: 3522.

[22] Thomas J , Harden A . Methods for the thematic synthesis of qualitative research in systematic reviews. BMC Med Res Methodol 2008; 8: 1–10. 18616818 10.1186/1471-2288-8-45 PMC2478656

[23] Dan B. Individuals with lived experience of disability should participate in every stage of research. Dev Med Child Neurol 2023; 65:4–5. 10.1111/dmcn.15438 36462202

[24] Coussens M , Destoop B , de Baets S , Desoete A , Oostra A , Vanderstraeten G , et al. A qualitative photo elicitation research study to elicit the perception of young children with developmental disabilities such as ADHD and/or DCD and/or ASD on their participation. PLoS One 2020; 15: 1–20. 10.1371/journal.pone.0229538 PMC7080235 32187183

[25] Krieger B , Piškur B , Schulze C , Beurskens A , Moser A . Environmental pre‐requisites and social interchange: the participation experience of adolescents with autism spectrum disorder in Zurich. Disabil Rehabil 2021; 43: 3789–802. 32356476 10.1080/09638288.2020.1753248

[26] Gantschnig BE , Hemmingsson H , La Cour K . Feeling and being involved? Participation experienced by children with disabilities at regular schools in Austria. J Occup Ther Sch Early Interv 2011; 4: 260–75.

[27] Conchar L , Bantjes J , Swartz L , Derman W . Barriers and facilitators to participation in physical activity: the experiences of a group of South African adolescents with cerebral palsy. J Health Psychol 2016; 21:152–63. 24607923 10.1177/1359105314523305

[28] Ullenhag A , Jahnsen R , Klove N , Smedvig S , Hoberg A . How did youth with cerebral palsy perceive participation in everyday life after participating in a periodical intensive rehabilitation program based on adapted physical activity in groups? A qualitative interview study. Disabil Rehabil 2024; 46: 58–66. 36803505 10.1080/09638288.2023.2180096

[29] Byhlin S , Käcker P . ‘I want to participate!’ Young adults with mild to moderate intellectual disabilities: how to increase participation and improve attitudes. Scand J Disabil Res 2018; 20: 172–81.

[30] Vänskä N , Sipari S , Haataja L . What makes participation meaningful? Using photo‐elicitation to interview children with disabilities. Phys Occup Ther Pediatr 2020; 40: 595–609. 32138590 10.1080/01942638.2020.1736234

[31] Pereira E , Cour K , Jonsson H , Hemmingsson H . The participation experience of children with disabilities in Portuguese mainstream schools. Br J Occup Ther 2010; 73: 598–606.

[32] Bantjes J , Swartz L , Conchar L , Derman W . Developing programmes to promote participation in sport among adolescents with disabilities: perceptions expressed by a group of South African adolescents with cerebral palsy. Intl J Disabil Dev Educ 2015; 62: 288–302.

[33] Nap‐Van Der Vlist MM , Kars MC , Berkelbach Van Der Sprenkel EE , Nijhof LN , Grootenhuis MA , van Geelen SM , et al. Daily life participation in childhood chronic disease: a qualitative study. Arch Dis Child 2020; 105: 463–9. 31748222 10.1136/archdischild-2019-318062

[34] Asbjørnslett M , Hemmingsson H . Participation at school as experienced by teenagers with physical disabilities. Scand J Occup Ther 2008; 15: 153–61. 19180721 10.1080/11038120802022045

[35] Devine MA . Leisure‐time physical activity: Experiences of college students with disabilities. Adapt Phys Activ Q 2016; 33: 176–94. 27078271 10.1123/APAQ.2014-0241

[36] Wintels SC , Smits DW , van Wesel F , et al. How do adolescents with cerebral palsy participate? Learning from their personal experiences. Health Expect 2018; 21:1024–34. 29858544 10.1111/hex.12796 PMC6250857

[37] Correia AM , Forlin C , Sio E . Exploring the participation and agency of students with special education needs in a Macau‐Chinese secondary school. J Res Spec Educ Needs 2022; 22: 288–96.

[38] Asbjørnslett M , Engelsrud GH , Helseth S . How children with disabilities engage in occupations during a transitional phase. J Occup Sci 2015; 22: 320–33.

[39] Veldhorst C , Wijnen M , Kef S , Vervloed MPJ , Steenbergen B . Participation of teenagers with vision or motor impairments in leisure activities: a qualitative study. Front Rehabil Sci 2024; 5: 1–15. 10.3389/fresc.2024.1444901 PMC11625804 39655184

[40] Renwick R , DuBois D , Cowen J , Cameron D , Fudge Schormans A , Rose N . Voices of youths on engagement in community life: a theoretical framework of belonging. Disabil Soc 2019; 34: 945–71.

[41] Huus K , Morwane R , Ramaahlo M , Balton S , Pettersson E , Berglund IG , et al. Voices of children with intellectual disabilities on participation in daily activities. Afr J Disabil 2021; 10: 1–9. 10.4102/ajod.v10i0.792 PMC8335764 34395201

[42] Brunes A , Krokstad E , Berit Augestad L . How to succeed? Physical activity for individuals who are blind. Br J Vis Impair 2017; 35: 264–74.

[43] Dahan‐Oliel N , Shikako‐Thomas K , Mazer B , Majnemer A . Adolescents with disabilities participate in the shopping mall: facilitators and barriers framed according to the ICF. Disabil Rehabil 2016; 38: 2102–13. 26729561 10.3109/09638288.2015.1114033

[44] Harding J , Harding K , Jamieson P , Mullally M , Politi C , Wong‐Sing E , et al. Children with disabilities' perceptions of activity participation and environments: A pilot study. Can J Occup Ther 2009; 76: 133–44. 19630303 10.1177/000841740907600302

[45] Harries T , Rettie R , Gabe J . Shedding new light on the (in)compatibility of chronic disease management with everyday life – social practice theory, mobile technologies and the interwoven time‐spaces of teenage life. Sociol Health Illn 2019; 41: 1396–409. 31124176 10.1111/1467-9566.12952

[46] Knibbe TJ , Biddiss E , Gladstone B , McPherson AC . Characterizing socially supportive environments relating to physical activity participation for young people with physical disabilities. Dev Neurorehabil 2017; 20: 294–300. 27715364 10.1080/17518423.2016.1211190

[47] Kreider CM , Bendixen RM , Mann WC , Young ME , Mccarty C . Mixed‐method Exploration of Social Network Links to Participation. OTJR 2015; 35: 151–9. 26594737 10.1177/1539449215578650 PMC4659346

[48] Lauruschkus K , Nordmark E , Hallström I . “It's fun, but …” Children with cerebral palsy and their experiences of participation in physical activities. Disabil Rehabil 2015; 37: 283–9. 24786968 10.3109/09638288.2014.915348

[49] Meier S , Höger B , Giese M . “If only balls could talk…”: barriers and opportunities to participation for students with blindness and visual impairment in specialized PE. Front Sports Act Living 2023; 5: 1–12. 10.3389/fspor.2023.1286909 PMC10754968 38162696

[50] Pontes TB , Mah K , Arnold AK , Polatajko HJ , Davis JA . The occupational repertoires of children with mobility difficulties: The child's perspective. Br J Occup Ther 2020; 83: 228–36.

[51] Volfson Z , McPherson AC , Tomasone JR , Faulkner GE , Arbour‐Nicitopoulos KP . Examining factors of physical activity participation in youth with spina bifida using the Theoretical Domains Framework. Disabil Health J 2020; 13: 1–7. 10.1016/j.dhjo.2020.100922 32312525

[52] Yeshanew YT , Xu T , Yuan W . Perceptions on their own social participation: A qualitative exploration of Ethiopian secondary students with visual impairments. Healthcare 2023; 11: 1–19. 10.3390/healthcare11040605 PMC9956288 36833141

[53] European Agency for Development in Special Needs Education . Five key messages for inclusive education ‐ putting theory into practice [Internet]. Odense: European Agency for Special Needs and Inclusive Education; 2014 [cited 2025 April 21]. 39p. Available from: https://www.european‐agency.org/sites/default/files/Five%20Key%20Messages%20for%20Inclusive%20Education.pdf

[54] Anaby D , Avery L , Gorter JW , Levin LF , Teplicky R , Turner L , et al. Improving body functions through participation in community activities among young people with physical disabilities. Dev Med Child Neurol 2020; 62: 640–8. 31670397 10.1111/dmcn.14382

[55] Maxwell G , Augustine L , Granlund M . Does thinking and doing the same thing amount to involved participation? Empirical explorations for finding a measure of intensity for a third ICF‐CY qualifier. Dev Neurorehabil 2012; 15: 274–83. 22646208 10.3109/17518423.2012.689780

[56] Csikszentmihalyi M. Flow. New York: HarperCollins Publishers; 1994.

[57] Kilgour G , Stott NS , Steele M , Adair B , Hogan A , Imms C . More than just having fun! Understanding the experience of involvement in physical activity of adolescents living with cerebral palsy. Disabil Rehabil 2024; 46: 3396–407. 37675880 10.1080/09638288.2023.2251395

[58] Ryan RM , Deci EL . Self‐determination theory and the facilitation of intrinsic motivation, social development, and well‐being. American Psychologist 2000; 55: 68–78. 11392867 10.1037//0003-066x.55.1.68

