# Supplementary material for: Involvement in daily life activities from the perspectives of children and young people with childhood‐onset disabilities: A scoping review
Source: Dev Med Child Neurol. 2026 Mar 12;68(10):1351–64. doi: 10.1111/dmcn.70228 (PMC13545496; doi:10.1111/dmcn.70228)
Supplement: Supplementary file 1 [file DMCN-68-1351-s001.pdf]

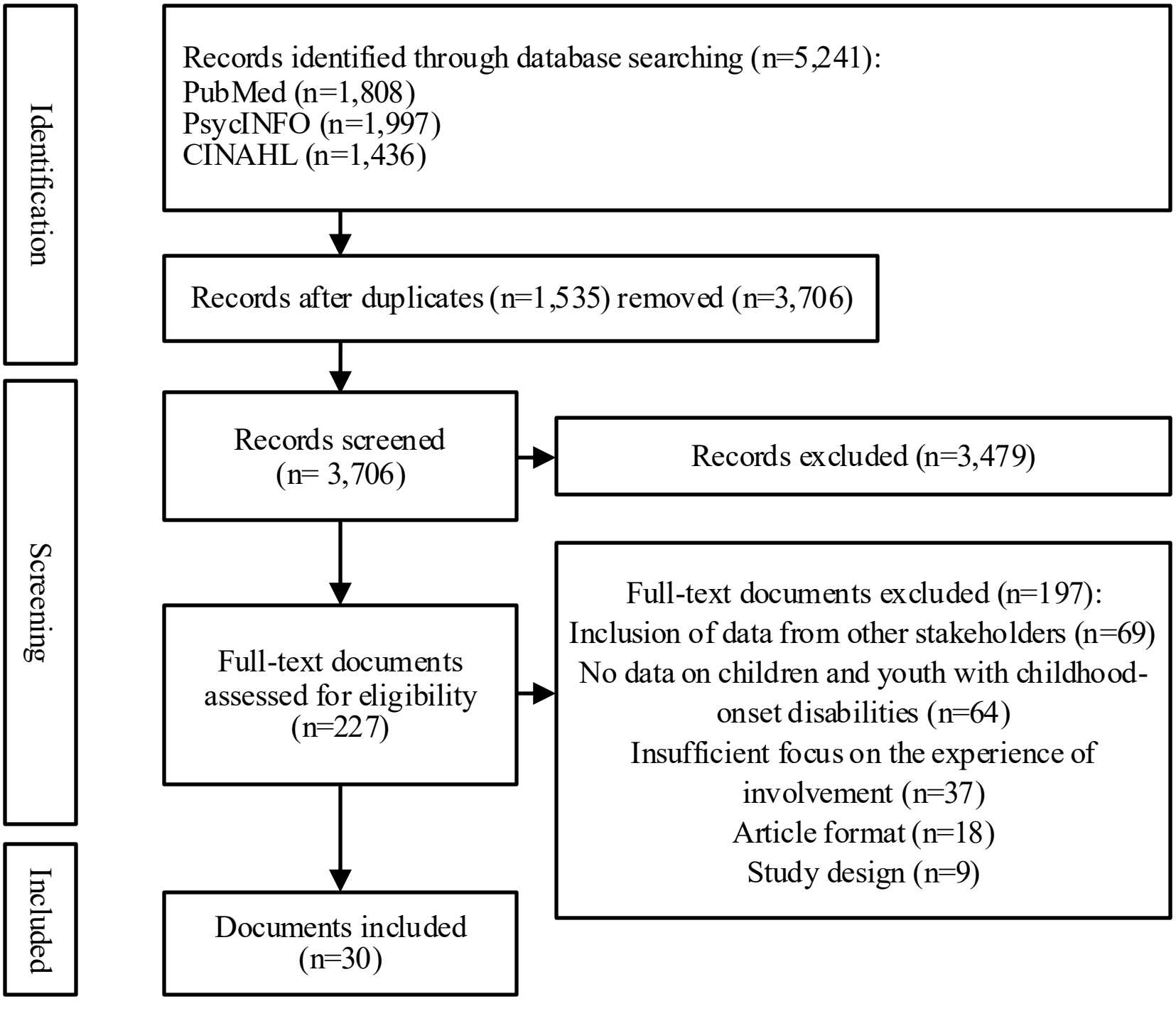
Identification
Screening
Included
Records identified through database searching (n=5,241):
PubMed (n=1,808)
PsycINFO (n=1,997)
CINAHL (n=1,436)
Records after duplicates (n=1,535) removed (n=3,706)
Records screened
(n= 3,706)
Records excluded (n=3,479)
Full-text documents
assessed for eligibility
(n=227)
Full-text documents excluded (n=197):
Inclusion of data from other stakeholders (n=69)
No data on children and youth with childhood-
onset disabilities (n=64)
Insufficient focus on the experience of
involvement (n=37)
Article format (n=18)
Study design (n=9)
Documents included
(n=30)
