# Supplementary material for: Involvement in daily life activities from the perspectives of children and young people with childhood‐onset disabilities: A scoping review
Source: Dev Med Child Neurol. 2026 Mar 12;68(10):1351–64. doi: 10.1111/dmcn.70228 (PMC13545496; doi:10.1111/dmcn.70228)
Supplement: Supplementary file 2 [file DMCN-68-1351-s002.docx]

**Appendix S1: Sample Search String**

**PubMed**

("child"[Mesh] OR "students"[mesh] OR "adolescent"[Mesh] OR "pediatrics"[Mesh] OR pediatric*[tiab] OR paediatric*[tiab] OR child*[tiab] OR student*[tiab] OR youth[tiab] OR teen*[tiab] OR adolescen*[tiab] OR "young adult"[tiab] OR “young adults”[tiab]) AND ("disabled persons"[Mesh] OR impair*[tiab] OR “chronic medical condition”[tiab] OR disab*[tiab] OR “special needs”[tiab]) AND (“social participation”[Mesh] OR "community participation"[Mesh] OR participation[tiab] OR involvement[tiab]) AND (“qualitative research”[Mesh] OR qualitative[tiab] OR interview*[tiab])

**CINAHL**

((MH "child") OR (MH "adolescence") OR (MH "students") OR (MH "pediatrics") OR AB("paediatric*") OR TI("paediatric*") OR AB("pediatric*") OR TI("pediatric*") OR AB("child*") OR TI("child*") OR AB("student*") OR TI("student*") OR AB("youth*") OR TI("youth*") OR AB("teen*") OR TI("teen*") OR AB("adolescen*") OR TI("adolescen*") OR AB("young adult") OR TI("young adult") OR AB("young adults") OR TI("young adults")) AND ((MH "Persons with Disabilities") OR AB("impair*") OR TI("impair*") OR AB("chronic medical condition") OR TI("chronic medical condition ") OR AB("disab*") OR TI("disab*") OR AB("special needs") OR TI("special needs")) AND ((MH "social participation") OR (MH "leisure participation") OR (MH "play participation") OR AB("participation") OR TI("participation") OR AB("involvement") OR TI("involvement")) AND ((MH "Qualitative Studies") OR AB("qualitative") OR TI("qualitative") OR AB("interview*") OR TI("interview*"))

**PsycINFO**

(DE "Pediatrics" OR DE "Elementary School Students" OR DE "Kindergarten Students" OR AB pediatric* OR TI pediatric* OR AB paediatric* OR TI paediatric* OR AB child* OR TI child* OR AB student* OR TI student* OR AB youth* OR TI youth* OR AB teen* OR TI teen* OR AB adolesc* OR TI adolesc* OR AB young adult OR TI young adult OR AB young adults OR TI young adults) AND (DE "Special Needs" OR DE "Disabilities" OR AB impair* OR TI impair* OR AB chronic medical condition OR TI chronic medical condition OR AB disab* OR TI disab* OR AB special needs OR TI special needs) AND (DE "Community Involvement" OR DE "Participation" OR AB participation OR TI participation OR AB involvement OR TI involvement) AND (DE "Qualitative Methods" OR AB qualitative OR TI qualitative OR AB interview* OR TI interview*)
